# Communication of support and critique in Swedish virtual community threads about prenatal diagnoses of fetal anomalies

**DOI:** 10.1186/s12884-016-0989-6

**Published:** 2016-07-29

**Authors:** Tommy Carlsson, Mats Landqvist, Elisabet Mattsson

**Affiliations:** 1 Department of Public Health and Caring Sciences, Uppsala University, BMC Husargatan 3, Box 564, S-75122 Uppsala, Sweden; 2 School of Culture and Education, Södertörn University, Stockholm, Sweden; 3 Department of Health Care Sciences, Ersta Sköndal University College, Stockholm, Sweden

**Keywords:** Internet, Pregnancy, Prenatal Diagnosis, Social Support

## Abstract

**Background:**

A prenatal diagnosis of a fetal anomaly involves acute grief and psychological distress. The Internet has the potential to provide virtual support following the diagnosis. The overall aim was to explore communication of support and critique in Swedish virtual community threads about prenatal diagnoses of fetal anomalies.

**Methods:**

Systematic searches in Google resulted in 117 eligible threads. Fifteen of these were purposefully selected and subjected to deductive content analysis.

**Results:**

The virtual support involved mainly emotional support (meaning units *n* = 1,992/3,688, 54 %) and was described as comforting and empowering. Posters with experience of a prenatal diagnosis appreciated the virtual support, including the opportunity to gain insight into other cases and to write about one’s own experience. Critique of the decision to continue or terminate the pregnancy occurred, primarily against termination of pregnancy. However, it was met with defense.

**Conclusions:**

Peer support, mainly emotional, is provided and highly appreciated in threads about prenatal diagnoses of a fetal anomaly. Critique of the decision to terminate the pregnancy occurs in virtual community threads about prenatal diagnoses, but the norm is to not question the decision. Future studies need to investigate if virtual peer support promotes psychosocial function following a prenatal diagnosis and what medium would be most suitable for these types of supportive structures.

## Background

Virtual communities (VC), namely “groups of people with common interests and practices that communicate regularly and for some duration in an organized way over the Internet through a common location or mechanism” [1], gather a growing number of people worldwide [2]. Benefits of computer-mediated communication include availability, anonymity, selective disclosure and social networking [3]. The Internet has the potential to offer distance-spanning peer support [4], that is social and emotional support mutually offered and provided by individuals sharing similar experiences [5]. Studies of computer-mediated communication have been conducted in several fields involving different health conditions [6–8], including reproductive subjects [9–11]. While the effects of virtual peer support remain inconclusive [4, 12, 13], prospective parents [4, 9, 13] and individuals who have experienced perinatal loss [11] appreciate virtual peer support. However, negative and even harmful aspects have also been reported among users of VC, for example polarization, illusions of well-being and increased prejudice [14].

Advances in prenatal screening have improved the detection rate of fetal anomalies during pregnancy [15–17]. Few are prepared for the diagnosis and the decision regarding whether to continue or terminate the pregnancy involves ethical [18] and informational difficulties [19, 20]. Women experience acute grief reactions and psychological distress following the diagnosis [21–23]. While termination of pregnancy following a prenatal diagnosis in many aspects is similar to other perinatal losses (i.e. stillbirth and miscarriage), it is different because it is a chosen loss [22]. Peer support is desired and appreciated following a prenatal diagnosis, both among those who continue [24] and those who terminate the pregnancy [25, 26]. However, research regarding virtual peer support and its potential value following a prenatal diagnosis is scarce.

### Theoretical framework

Four theory-driven attributes of social support have previously been identified: affirmational, emotional, informational and instrumental [27, 28]. *Affirmational support* is communication that affirms emotions, cognitions and behaviors of the recipient and motivates the recipient to solve problems and gain optimism [28]. *Emotional support* is communication of caring and concern conveyed, for example, by listening/reading, reassuring, comforting and empathizing, and helps restore self-esteem and reduces distress and feelings of inadequacy. *Informational support* is communication of information to guide or advise, intended to improve control, reduce confusion, and increase optimism about the future [29]. As this study investigates virtual support, *instrumental support,* typically involving practical support [28], will here be defined as offers of personal support outside the thread, either as personal communication through online messages or in face-to-face settings.

### Aim

The overall aim was to explore communication of support and critique in Swedish virtual community threads about prenatal diagnoses of fetal anomalies. The following research questions were addressed:

1. How is affirmational, emotional, informational and instrumental virtual support distributed and communicated?
2. How is the value of virtual support distributed and described?
3. What critiques are expressed regarding the decision to continue or terminate the pregnancy and how do others respond to these reactions?

## Methods

### Search procedure

**Fig. 1:**
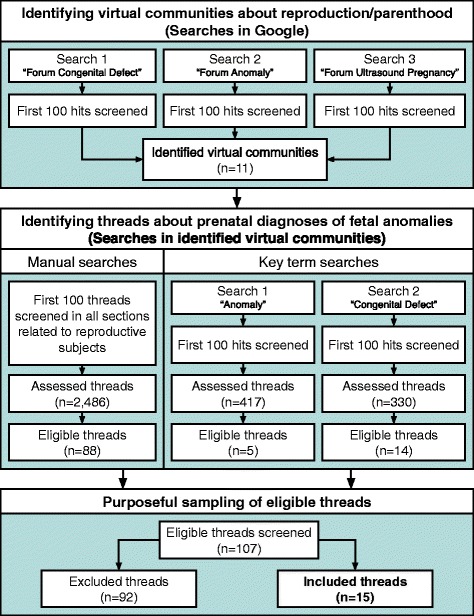
The sampling procedure

The process of including threads started with systematic searches to identify VC threads, followed by purposeful selection of identified threads. Figure 1 presents the sampling procedure.

#### Identifying virtual communities about reproduction/parenthood

Three searches were conducted in Google, the most used search engine on the Internet [30], to find host websites for Swedish VC about reproduction/parenthood. The searches were performed in June 2014 using the key terms “Forum Congenital Defect”, “Forum Anomaly” and “Forum Ultrasound Pregnancy”. Since the searches resulted in hits ranging from 54,100 and 345,000, it was impossible to screen all hits. Thus, the first 100 hits of each search were screened for Swedish VC about reproduction/parenthood. In total, 11 VC were identified.

#### Identifying threads about prenatal diagnoses of fetal anomalies

The identified VC were subjected to manual and key term searches to identify threads about prenatal diagnoses of fetal anomalies. Manual searches involved screening of the first 100 threads in all sections related to reproductive subjects. Key term searches were performed when possible and involved screening of the first 100 hits using the terms “Anomaly” and “Congenital Defect”. The searches yielded 107 eligible threads, i.e. with a thread-starter that described experience of a pregnancy where a fetal anomaly was detected.

#### Purposeful sampling of eligible threads

A qualitative analysis of all threads was not considered feasible. Thus, the first author conducted a purposeful sampling [31] to select threads with maximum variation regarding background of the posters, type of fetal anomaly, termination or continuation of the pregnancy, and subjects covered in the threads. The purposeful selection was performed until saturation was considered achieved. In total, 15 threads were included in the study.

### Sample characteristics

The threads were initiated between 2006 and 2014, included 3,126 messages (range = 11–1,143), were written by 349 posters (range = 3–93) and contained 313,169 words (range = 618–109,700). The majority of the posters presented themselves as females (*n* = 229, 66 %), whereas 119 (34 %) did not present their sex and one poster (0.3 %) presented himself as male. Half of all posters had experience of a congenital anomaly (*n* = 176). Of the posters with experience of a prenatal diagnosis (*n* = 148, 42 %), 125 (84 %) terminated the pregnancy, 20 (14 %) continued the pregnancy, and three (2 %) did not disclose their decision. Table 1 presents the backgrounds of the posters.

**Table 1:** Backgrounds of the posters (*n* = 349)

Background^a^	N(%)
Not disclosed/No personal experience of congenital anomaly	173(50)
Termination of pregnancy following a fetal anomaly	108(31)
Parent/relative to child with congenital anomaly	37(10)
Currently pregnant with prenatal diagnosis	28(8)
Person with congenital anomaly	3(1)

^a^When first writing in the thread

Table 2 presents type of congenital anomaly among the posters with presented experience of a prenatal or postnatal diagnosis (n=176). The most common anomalies were chromosomal (*n* = 47, 27 %) and heart defects (*n* = 35, 20 %).

**Table 2:** Type of congenital anomaly among the posters with presented experience of a prenatal or postnatal diagnosis (n=176)

Type of anomaly	n(%)
Chromosomal	47(27)
Heart defect	35(20)
Multiple	31(18)
Not disclosed	27(15)
Brain	12(7)
Kidney and urinary tract	12(7)
Spina bifida	9(5)
Club foot	1(1)
Congenital amputation	1(1)
Tumor	1(1)

### Data analysis

The selected threads were cleaned of advertisements and subjected to qualitative content analysis, a method to describe patterns in data [32]. The first author, who performed the initial coding, kept a reflective journal during the data collection and analysis [31]. To get familiarized with the material, the threads were read repeatedly. Guided by the theoretical framework, meaning units (MU) were deductively identified according to the theoretical framework and the research questions. An MU was defined as words, sentences or paragraphs containing aspects related to each other through content and context. Table 3 presents an example of the process of identification and coding of MU.

**Table 3:** Examples of identification and coding of meaning units

Original message	Identified meaning unit	Code
Had a look at your thread and was horrified by what incredibly stupid and callous people there are. You should absolutely not bother about them. You are a fantastic person and you will get through this. Sending you strength! Hugs <3	Had a look at your thread and was horrified by what incredibly stupid and callous people there are. You should absolutely not bother about them.	Response to critique
	You are a fantastic person and you will get through this.	Affirmational support
	Sending you strength! Hugs <3	Emotional support

By reviewing the raw material, MU, and reflective journal, commonalities were explored and identified together with the second and last author. Joint discussions among the authors were held until consensus was reached. Nvivo for Mac (version 10.2.0, QSR International, Doncaster, Victoria, Australia) was utilized to conduct the qualitative analysis. The data collection and analysis were concurrent and finished when saturation was considered achieved [31]. Descriptive statistics were analyzed with R (version 3.1.1).

## Results

### Distribution and communication of affirmational, emotional, informational and instrumental virtual support

Table 4 presents distribution of MU and illustrative quotes of affirmational, emotional, informational and instrumental support. The majority of the MU related to communication of virtual support was emotional (MU *n* = 1,992, 54 %), whereas informational (MU *n* = 812, 22 %) and affirmational (MU *n* = 807, 22 %) were less common. Instrumental support was rare (MU *n* = 77, 2 %). Across all support attributes, posters who were currently pregnant when first writing in the thread and those with previous experience of termination of pregnancy following a fetal anomaly wrote the majority of the MU related to communication of virtual support.

**Table 4:** Distribution of meaning units (MU) and illustrative quotes of affirmational (MU *n* = 807), emotional (MU *n* = 1,992), informational (MU *n* = 812) and instrumental (MU *n* = 77) support

Support attribute	Background of poster^a^	MU n(%)	Illustrative quote
Affirmational (MU *n* = 807, 21.9 %)	Currently pregnant with prenatal diagnosis	317(39.3)	*You sure haven’t had an easy time…*
	Termination of pregnancy following a fetal anomaly	294(36.4)	*Small comfort when the worry flashes through your body, I know…*
	Not disclosed/No personal experience of congenital anomaly	127(15.8)	*You are strong and selfless doing this for the sake of your child!*
	Parent/relative to child with congenital anomaly	69(8.5)	*I can understand that it feels tough right now*
Emotional (MU *n* = 1,992, 54.0 %)	Termination of pregnancy following a fetal anomaly	890(44.7)	*Hugs to you*
	Currently pregnant with prenatal diagnosis	670(33.6)	*Here come lots of warm fortifying hugs for today*
	Not disclosed/No personal experience of congenital anomaly	303(15.2)	*I wish it was possible to send strength through the computer… <3*
	Parent/relative to child with congenital anomaly	128(6.4)	*supportive hugs* and *supportive thoughts*
	Person with congenital anomaly	1(0.1)	*Sure everything will be OK. Luck seems to be on your side*
Informational (MU *n* = 812, 22.0 %)	Termination of pregnancy following a fetal anomaly	333(41.0)	*Took some photos (TAKE A CAMERA)*
	Currently pregnant with prenatal diagnosis	225(27.7)	*There’s only a 50 % chance of a “CDH” baby reaching its first birthday*
	Parent/relative to child with congenital anomaly	140(17.3)	*Today they can save children born in week 22*
	Not disclosed/No personal experience of congenital anomaly	113(13.9)	*There are only two children’s heart centers in the country*
	Person with congenital anomaly	1(0.1)	*I’ve had operations on the heart defect I was born with 3*
Instrumental (MU *n* = 77, 2.1 %)	Termination of pregnancy following a fetal anomaly	35(45.4)	*If anything crops up, don’t hesitate to get in touch.*
	Currently pregnant with prenatal diagnosis	24(31.2)	*If you feel a need to talk to someone […] just let me know.*
	Not disclosed/No personal experience of congenital anomaly	10(13.0)	*So we can take a trip and have a coffee together?*
	Parent/relative to child with congenital anomaly	8(10.4)	*If you live close by, perhaps we can meet and talk.*

^a^When first writing in the thread

#### Affirmational support

Drawing from their own experiences, posters with experience of a prenatal diagnosis affirmed the painful and difficult situation following the diagnosis. Through their messages, posters with and without experience of a congenital anomaly validated the thoughts and feelings of posters faced with a recent diagnosis.

#### Emotional support

A number of different emoticons were used to provide emotional support, such as :-) and <3. Virtual hugs were frequently used to reach through the web and offer distance-spanning emotional support. Messages of encouragement and reassurance were written to posters who were faced with a difficult situation as time went on, for example the abortion procedure and calculated due date when terminating the pregnancy, and follow-up prenatal tests and the birth when continuing the pregnancy. The emotional support also included condolences, for example regarding the fact that they had received a prenatal diagnosis, had to terminate the pregnancy, or if the fetal defect had worsened during the course of the pregnancy when it was continued.

#### Informational support

Informational support often included a combination of communication of facts and personal experiences. The informational support included information about emotional difficulties, decision-making about continuation or termination of pregnancy, medically induced abortions (e.g. pain relief and seeing the fetus), the postpartum period (e.g. symptoms and treatments of the anomaly), prenatal tests/risk of recurrence in future pregnancies, strategies to become pregnant again following an abortion, and managing through the healthcare system.

#### Instrumental support

Posters offered personal communication through messages and face-to-face meetings. Some wanted to interact in person rather than via the Internet and actively searched for persons to meet in face-to-face settings. Others seemed to prefer online interactions exclusively. While offers of face-to-face meetings occurred, we did not see any evidence that these ever took place.

### Distribution and described value of virtual support

Table 5 presents distribution of MU and illustrative quotes of described value of virtual support. The majority of the MU related to described value of virtual support (MU *n* = 343) were written by posters who were pregnant when first writing in the thread (MU *n* = 192, 56 %) and with experience of termination of pregnancy (MU *n* = 133, 39 %).

**Table 5:** Distribution of meaning units (MU) and illustrative quotes of described value of virtual support (MU *n* = 343)

Background of poster^a^	MU n(%)	Illustrative quote
Currently pregnant with prenatal diagnosis	192(56.0)	*That’s why this thread I’ve created is so great, as we had decided that that I can shout and cry and feel sorry for myself here […] You are all my angels!!*
Termination of pregnancy following a fetal anomaly	133(38.8)	*Thanks for your support. The tears just run down my cheeks when I read everything.*
Not disclosed/No personal experience of congenital anomaly	16(4.6)	*When I read what you have written, I realize what strong people there are out there. It gives me strength too.*
Parent to child with congenital anomaly	2(0.6)	*Thanks for your kind and encouraging words!*

^a^When first writing in the thread

The posters expressed appreciation of the opportunity to gain insight into the life of peers with similar experiences, empowering them to move forward and find strength. In particular, the posters described comfort when reading about the experiences of others, which eased loneliness and validated feelings. To know that others had moved on and recuperated provided a sense of security, comfort, and strength.

*It feels good that there are more people out there who have been through the same thing as me and who have moved on, who have been able to have children again and it gives me strength.*

Although many posters expressed appreciation of professional psychosocial support, virtual peer support was described as different as the peers had real life experiences of a prenatal diagnosis, in comparison to professionals.

*Yes, you and the thread are invaluable. Talking to a psychologist, for example, is good, I’m sure, but they nod and say everything is normal. But I would have felt that they had to say that. But when real people write that they feel the same and you can recognize yourself in what they write, it feels better.*

The posters expressed appreciation of the opportunity to write about their own experiences, which was a way to cope and reflect on the situation, for example to “complain a bit” and “ventilate thoughts”.

*Without you here I’d have gone nuts!! Air loads of thoughts and feelings! You feel so terrible but then you write here and then you don’t feel so bad any more.*

### Critique of continuation/termination of pregnancy and responses

#### Voiced critique

In total, 36 MU of critique were identified. The majority of these were against termination of pregnancy (MU *n* = 30, 83 %) and only expressed by posters that did not disclose any experience of a prenatal diagnosis. In some threads it was clear that moderators had deleted offensive posts. Primarily, the critique of termination of pregnancy involved ending a life and not giving the fetus a chance to live.

*I think it’s terrible to give birth to the child after week 20, let it lie there and die because the child MAYBE won’t survive if it goes full term. What’s humane about that?*

All critique of continuation of pregnancy originated from posters of non-disclosed backgrounds. The critique involved putting a disabled child into the world, causing it unnecessary suffering and possibly death.

*I would never have given birth to a handicapped child. MEAN!*

#### Responses to critique

In total, 142 MU of responses to critique were identified, all defending the decision to terminate the pregnancy. The majority of these were written by posters with experience of termination of pregnancy (MU *n* = 64, 45 %) and with non-disclosed backgrounds (MU *n* = 44, 31 %).

A distinct norm in the threads was that the decision to terminate the pregnancy was not to be questioned. When critique was voiced, posters with and without experience of a prenatal diagnosis reacted to protect peers. Posters who had terminated the pregnancy defended their decision, describing it as personal and carefully considered. They asked others to respect the decision and not write critique, defending the thread as a medium to offer support.

*If TS’s [Thread starter] decision annoys you, then you don’t need to say anything! Everyone has different opinions, that’s just how it is, but it’s quite wrong to express them here as they hurt and don’t help TS or anyone else.*

## Discussion

This study explored communication of support and critique in virtual community threads about prenatal diagnoses of fetal anomalies and found that the majority of the support was emotional, whereas informational and affirmational support was less common and instrumental support rare. Described value of the virtual support included gaining insight into other cases and the opportunity to write about one’s own experience. Critique of the decision to terminate the pregnancy occurred, but was met with defense, as the norm was that others should not question the decision.

Four attributes of peer support have previously been identified in theory: affirmational, emotional, informational and instrumental support [27, 28]. While all four of these different attributes could be identified in the threads, the emphasis and most common was emotional and not informational support. It is thus possible that informational support is mostly gained through other mediums than VC, for example via health professionals and information websites. Results from this study suggest that virtual peer support fills other needs than professional support.

Previous research indicates that social support is of importance following a prenatal diagnosis [24, 33] and perinatal loss [34, 35]. The posters in this study appreciated the opportunity to connect with hard-to-reach peers and write about their own experiences. It has been put forward that self-disclosure through written emotional expression has cognitive and social benefits [36], and improves health outcomes in different settings [37, 38]. Because of the distance-spanning capabilities of the Internet, VC may serve as a tool for emotional expression and peer support in vulnerable situations. Even though the effects of virtual peer support remain inconclusive [4, 12, 13], the findings indicate it is beneficial following a prenatal diagnosis, which is in line with a previous study about perinatal loss [11]. Although not captured here, it is important to bear in mind that virtual peer support may also be associated with negative aspects, for example decreased face-to-face interactions [39] and excessive reliance on virtual support groups [40].

In this study a distinct norm in the threads was that the decision to terminate the pregnancy was personal and should not be questioned by others. Thus, the previously reported stigma surrounding termination of pregnancy [41, 42] was challenged in the threads. It could be hypothezied that VC offer a place to express feelings and get support from peers following an abortion due to a fetal anomaly. Nevertheless, critique of termination of pregnancy occurred, which indicates that virtual peer support among those who terminate the pregnancy might be most suited for non-public settings. More research is needed to investigate the most appropriate medium to offer peer support in this context.

Taken together, we identified considerable peer support in the threads. Advantages of engagement in VC - either as a supporter, a recipient of support, or both - include altruism, a reduced sense of isolation, and being part of a nonjudgmental community [43]. The helper therapy principle [44] may further explain the findings, suggesting that support is best received when the context offers no stigma and when receivers have direct control over the support, are members of self-help mutual aid groups, and are similar to the helpers. All of these factors could be applied to the included VC. Furthermore, the theory [44] suggests that professional support is embedded with a natural asymmetrical relationship where the helpee is in a dependent role associated with lesser status than the helper. This in turn may explain the fact that the posters described appreciation of professional psychosocial support following the diagnosis, but also distinguished between professional and virtual peer support. The findings verify previous reports that each kind of support meets different needs and cannot replace one another [45]. Thus, the findings suggest that, in this context, support from peers might have different positive benefits than those gained from professional psychosocial support. The coexistence of these two support systems may further facilitate psychosocial function after the diagnosis.

### Methodological considerations

This study aimed to explore virtual peer support in public Internet forums and thus only concerns individuals with access to the Internet. Purposeful sampling was used to select threads with maximum variation. Despite this, only 20 posters presented continuation of pregnancy following a prenatal diagnosis, and only one presented himself as male. It is reasonable to assume that these groups either use other mediums to communicate with peers, or that the use of VC is less in these particular groups.

To identify relevant VC about reproduction/parenthood, we conducted three searches in Google and screened the first 100 hits. Thus, it is possible that we failed to identify all relevant VC. However, the included VC were large and active. To identify relevant threads, we conducted both manual and key term searches, screening 3,233 threads. Consequently, we argue that the search procedure was rigorous and comprehensive. Fifteen threads identified by purposeful sampling from 117 threads were included in this study, determined through data saturation. This strengthens the transferability of the findings. However, we acknowledge that experiences of a prenatal diagnosis are dependent on the situational context, for example routine ultrasound screening and current state laws concerning the possibility to terminate the pregnancy. Considering that distinct data saturation was achieved from a large amount of posters and messages, we find it reasonable to assume that the findings are transferable to settings with legislation and prenatal care that are similar to Sweden.

This study used covert methods to collect archived data. Thus, no interviews or member checks were conducted. This fact could possibly imply that the results do not fully reflect the posters’ individual experiences. It does, however, reflect the material as presented through the threads and should be interpreted with this in mind. The fact that archived asynchronous data was used and that the researcher remained covert during data collection strengthens the internal reliability of the study, since it was possible to collect data without any potential researcher influence [46]. As with all qualitative studies, the analysis is the product of the perspectives and observations of the researcher [31]. Thus, the first author who conducted data collection and primary analysis influenced the results through his background, for example his professional background as a nurse. To approach this, a reflective journal was kept to encourage reflexivity, i.e. self-awareness of cultural, political, social, linguistic, and ideological perspectives [31]. Furthermore, the second and last authors were involved in the later stages of analysis to achieve consensus between several researchers.

### Suggestions for future research

More research is needed regarding the impact on the psychosocial well-being of peers giving and receiving virtual support following a prenatal diagnosis. Future studies should adopt experimental and longitudinal designs to investigate how the support translates into psychosocial and healthcare outcomes. More research is also needed to investigate the most appropriate medium to offer peer support in this context. Furthermore, future studies should explore the trustworthiness and accuracy of informational support communicated in VC.

This study did not take into account possible lurkers, i.e. readers of threads who never posted any messages. Previous research suggests that lurkers within health-related computer-mediated communications are a substantial part of membership [47] and that lurking in VC may have beneficial effects, in particular concerning advice and insight [48]. Thus, more research is needed regarding lurkers in VC addressing prenatal diagnosis.

## Conclusion

Peer support, mainly emotional, is provided and highly appreciated in threads about prenatal diagnoses of a fetal anomaly. Critique of the decision to terminate the pregnancy occurs in virtual community threads about prenatal diagnoses, but the norm is to not question the decision. Future studies need to investigate if virtual peer support promotes psychosocial function following a prenatal diagnosis and what medium would be most suitable for these types of supportive structures.

## Abbreviations

CHD, congenital heart defects; MU, meaning units; VC, virtual communities
